# Supplementary material for: Genome-Wide Transcriptional Profiling Reveals PHACTR1 as a Novel Molecular Target of Resveratrol in Endothelial Homeostasis
Source: Nutrients. 2022 Oct 27;14(21):4518. doi: 10.3390/nu14214518 (PMC9658213; doi:10.3390/nu14214518)
Supplement: Supplementary file 1 [file nutrients-14-04518-s001.zip › nutrients-1960364-Supplemental Materials.pdf]

Supplemental Materials

Supplemental Table S1. Primer sequence.

**Primers**	**Sequences**
hKLF2-S	GCCGTCCTTCTCCACTTTC
hKLF2-AS	GAAGTCCAGCACGCTGTT
hEDN1-S	AAGGCAACAGACCGTGAAA
hEDN1-AS	GTCTTCAGCCCTGAGTTCTTT
hPHACTR1-S	GGAACTGGAACAGAGGAACAT
hPHACTR1-AS	TTGACTGAGCTTTCGGGTTAG
hGAPDH-S	GATTCCACCCATGGCAAATTC
hGAPDH-AS	CTGGAAGATGGTGATGGGATT

Supplemental Table S2. Antibodies and sources.

**Antibody**	**Catlog# and Vendor**
PHACTR1	#ab229120, Abcam
GAPDH	#60004-1-Ig, Proteintech
